# Phorbol Myristate Acetate Inhibits Senecavirus A Replication by Activating IKBKE–Mediated IFN Pathway and NF‐κB Signal

**DOI:** 10.1155/tbed/5583480

**Published:** 2026-01-31

**Authors:** Junfang Yan, Yanni Gao, Chengyi Guo, Yubei Dong, Ping Jiang, Juan Bai

**Affiliations:** ^1^ Key Laboratory of Animal Diseases Diagnostic and Immunology, Ministry of Agriculture, MOE International Joint Collaborative Research Laboratory for Animal Health and Food Safety, College of Veterinary Medicine, Nanjing Agricultural University, Nanjing, 210095, China, njau.edu.cn; ^2^ Key Laboratory of Applied Biotechnology on Animal Science and Veterinary Medicine of Zhejiang Province, Zhejiang Engineering Research Center for Veterinary Diagnostics and Advanced Technology, Zhejiang International Science and Technology Cooperation Base for Veterinary Medicine and Health Management, Belt and Road International Joint Laboratory for One Health and Food Safety, China-Australia Joint Laboratory for Animal Health Big Data Analytics, College of Veterinary Medicine of Zhejiang A&F University, Hangzhou, Zhejiang, China; ^3^ Jiangsu Co-Innovation Center for Prevention and Control of Important Animal Infectious Diseases and Zoonoses, Yangzhou, China, yzu.edu.cn

**Keywords:** IKBKE, immune response, phorbol myristate acetate, Senecavirus A

## Abstract

Senecavirus A (SVA) is an emerging picornavirus causing vesicular disease indistinguishable from foot‐and‐mouth disease virus (FMDV). So far, there are no commercial vaccines and effective therapeutic drugs against SVA infection in China. Here, a library of 112 compounds were screened, and we found that phorbol myristate acetate plays an antagonistic role in the early stage of SVA infection. And phorbol 12‐myristate 13‐acetate (PMA) upregulates the expression of IKBKE, and activates IFN pathway and NF‐κB signal. However, the PMA–mediated detrimental effect on SVA is reversed in IKBKE–deficient cells or when the NF‐κB pathway blocked by BAY‐117082, implying that IKBKE is the target for the antiviral effect of PMA. Additionally, PMA possesses antiviral effect on multiple RNA viruses, including porcine epidemic diarrhea virus (PEDV), porcine reproductive and respiratory syndrome virus (PRRSV), and encephalomyocarditis virus (EMCV). Overall, our findings offer that PMA inhibits SVA replication by activating IKBKE–mediated IFN pathway and NF‐κB signal. And it might be a promising candidate for further broad‐spectrum therapeutic development.

## 1. Introduction

Formerly known as Seneca Valley virus, Senecavirus A (SVA) is a nonenveloped and single‐stranded RNA virus belonging to family Picornaviridae [1]. SVA remains epidemic in countries around the world such as Canada, Brazil, the United States, Colombia, Thailand, and China [2–7]. Characteristic lesions associated with SVA infection include vesicles on the snout, oral mucosa, hoofs, and coronary bands, which are indistinguishable from foot‐and‐mouth disease virus (FMDV), vesicular stomatitis virus (VSV), swine vesicular disease virus (SVDV), and vesicular exanthema of swine virus (VESV), causing a significant economic loss to the pig industry [2, 6, 8–10]. Hence, it is necessary to carry out strategies to prevent and control SVA infection.

Vaccination plays an important role in the prevention and control of animal infectious diseases. Currently, inactivated or attenuated vaccines are considered the most effective measures to prevent SVA infection [11–15]. However, the continuous and long‐term circulation, transmission, and evolution of SVA isolates have hindered the screening of vaccine candidates. In addition, high cost of the pig model for vaccine evaluation is also a substantial obstacle. Therefore, there is an urgent need to develop alternative antiviral strategies to combat the virus. Recently, antiviral drugs represent the mainstay for preventing SVA including bovine lactoferricin‐lactoferrampin (LFCA), PFD5 (4‐chloro‐2‐(5‐phenyl‐1‐(pyridin‐2‐yl)‐4,5‐dihydro‐1h‐pyrazol‐3‐yl)phenyl), Grp94 Inhibitor HCP1, monolaurin, and MSA‐2 [16–20]. No commercialized compounds against SVA have been approved to date. To identify SVA antagonist, 112 small molecules were screened, and phorbol‐12‐myristate‐13‐acetate (PMA) with high antiviral activity was found.

Mechanically, IKBKE expression was upregulated, the IFN pathway was activated and the NF‐κB cascade was amplified in PMA–treated cells. The inhibition rate of SVA replication by PMA was reduced in IKBKE‐deficient cells, and PMA mediated adverse effect on SVA was eliminated when the NF‐κB pathway was blocked by BAY‐117082. Importantly, PMA exhibits broad‐spectrum antiviral properties, which provides new insights for subsequently development of antiviral therapeutic targets.

## 2. Results

### 2.1. Screening of Small Molecular Compounds Against SVA Infection

To develop an alternative antiviral drug to prevent and control infection of SVA, we investigated the antiviral effects of 112 compounds, including 87 natural products and 25 chemical agents. The processing and identification diagram is shown in Figure 1A,B. As previous described [21, 22], PK‐15 cells were treated with 10 µM compounds and infected with SVA. We preliminarily screened the library by indirect immunofluorescence (Figure 1C). Fortunately, eight natural products and two chemicals inhibited SVA proliferation (Figure 1D). These compounds were subjected to a second round of screening by western blot (Figure 1E). And the antiviral activity of PMA, raddeanin A, mubritinib, and polyphyllin II were repeatedly validated (Figure 1F). In order to further explore the effects of the four compounds mentioned above on SVA transmission, PK‐15 cells were treated with different concentrations of compounds. We found a positive correlation between compound concentration and antiviral activity (Figure 2). In addition, the 50% cytotoxic concentration (CC_50_) and the 50% inhibition concentration (IC_50_) were determined by CCK‐8 kit and IFA, respectively (Figure 2A–D). And, the selection index (SI) of PMA was the highest (Figure 2E). Together, the date show that PMA has potential development value on antagonizing SVA infection.

Figure 1Screening of compounds against SVA infection. (A) Diagram showing drugs treatment and virus inoculation. PK‐15 cells were pretreated with 10 µM compounds for 1 h and then infected with SVA (1 MOI) for 1 h. Cells were washed with PBS and then incubated for another 24 h in medium containing 10 µM compounds. (B) Screening process flowchart. (C) The primary screening was performed by indirect immunofluroescence method. Each dot represents the percentage of SVA (1 MOI) replication inhibited by each compound (10 µM). (D) Preliminary screening results of compound library. The compounds listed in the table showed no obvious cytotoxicity and the viral inhibition rate was higher than 50%. (E) The antiviral effects of the above compounds were identified by western blot. (F) Confirmatory screening results of compound library.

**Figure:**
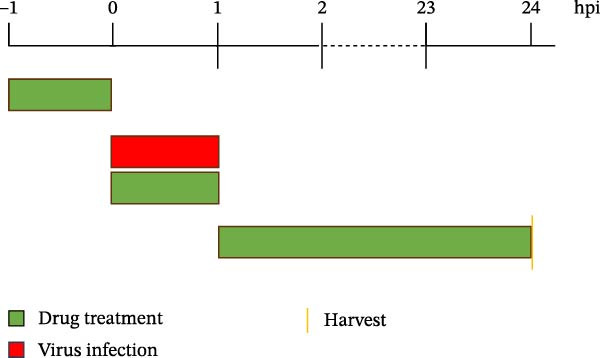
(A)

**Figure:**
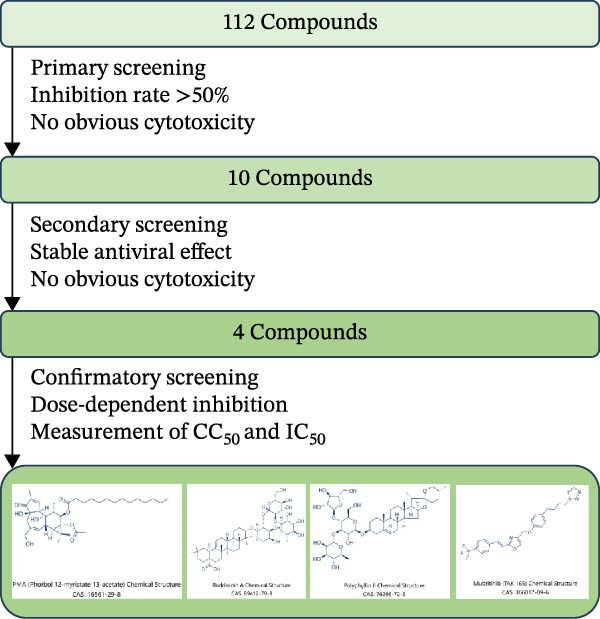
(B)

**Figure:**
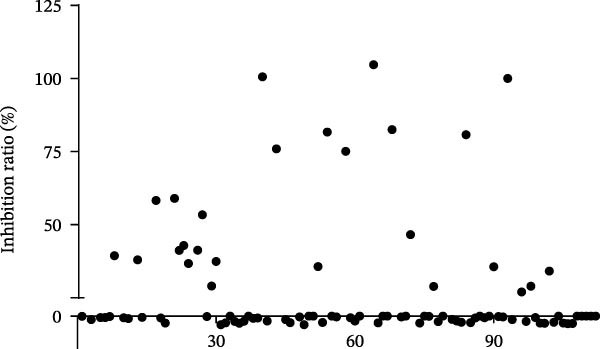
(C)

**Figure:**
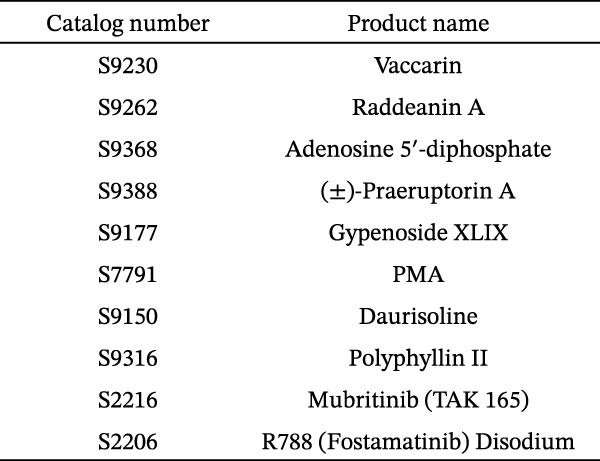
(D)

**Figure:**
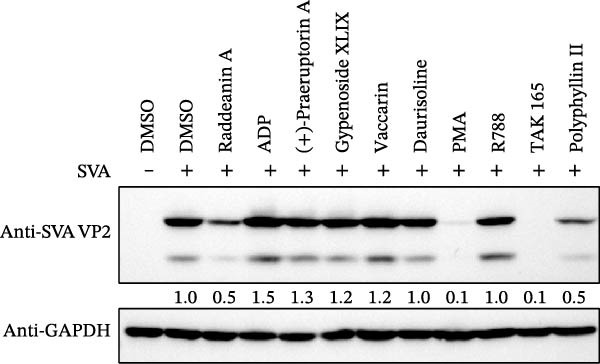
(E)

**Figure:**
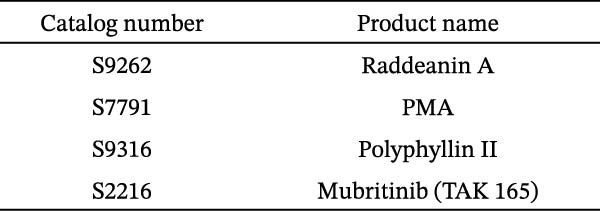
(F)

Figure 2Characterization of four compounds against SVA infection. (A) CC_50_ and IC_50_ curves of raddeanin A. (B) CC_50_ and IC_50_ curves of polyphyllin II. (C) CC_50_ and IC_50_ curves of TAK‐165. (D) CC_50_ and IC_50_ curves of PMA. (E) Selectivity index (SI) of the four designated compounds. SI = CC_50_/IC_50_.

**Figure:**
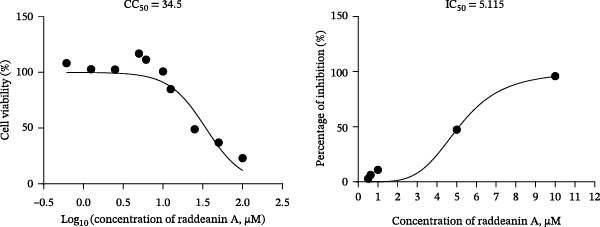
(A)

**Figure:**
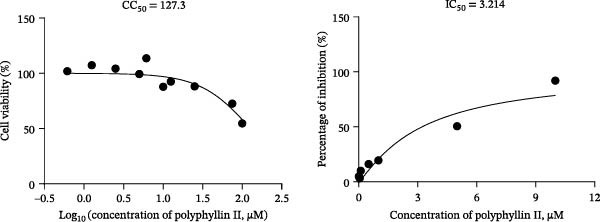
(B)

**Figure:**
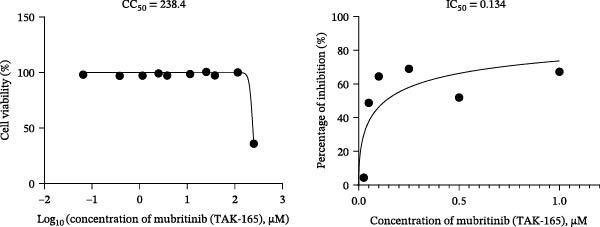
(C)

**Figure:**
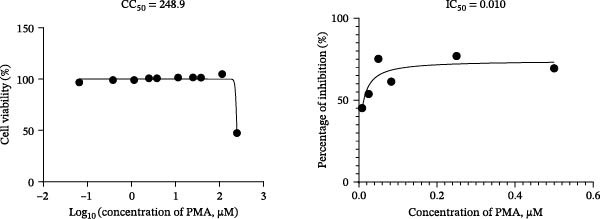
(D)

**Figure:**
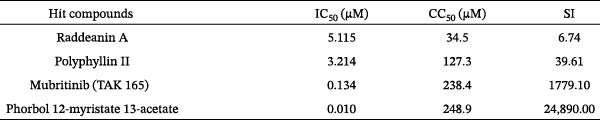
(E)

### 2.2. PMA Inhibits SVA Replication

In view of the high SI of PMA, we elaborated the inhibition of PMA on SVA from multiple dimensions. PK‐15 cells were treated with DMSO or PMA and infected with SVA at multiplicity of infection (MOI = 1) for 1 h. At 24 hpi, cells were collected for western blot. PMA inhibits SVA replication in a dose‐dependent manner (Figure 3A). The cells were fixed and permeabilized for IFA assay. With the increase of compound concentration, the number of green spots dramatically decreased (Figure 3B). Similarly, viral RNA levels were quantitated using qRT‐PCR (Figure 3C). Correspondingly, progeny virus production was also significantly downregulated (Figure 3D). Collectively, the results suggest that PMA is a negative regulator of viral replication.

Figure 3PMA inhibits SVA replication in a dose‐dependent manner. PK‐15 cells were pretreated with 10 µM compounds for 1 h and then infected with SVA (1 MOI) for 1 h. Cells were washed with PBS and then incubated for another 24 h in medium containing 10 µM compounds. Cells were harvested for western blot (A), IFA assay (B), qRT‐PCR (C), and TCID_50_ (D), respectively. All samples run in triplicate. ns, not significant;  ^∗^, *p*  < 0.05;  ^∗∗^, *p*  < 0.01,  ^∗∗∗^, *p* < 0.001.

**Figure:**
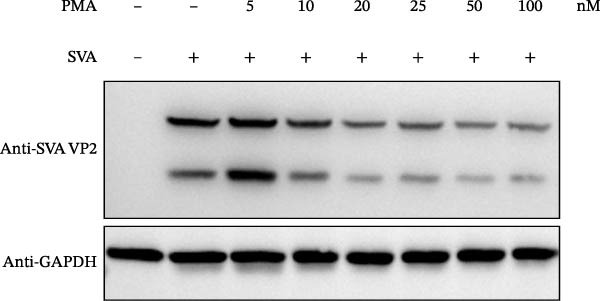
(A)

**Figure:**
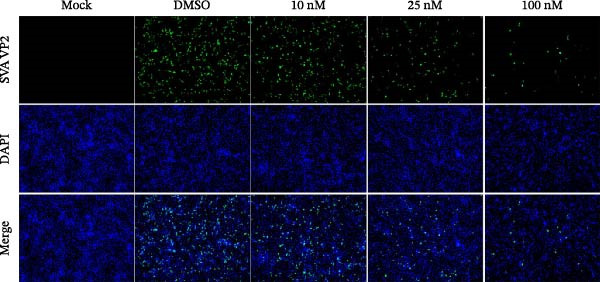
(B)

**Figure:**
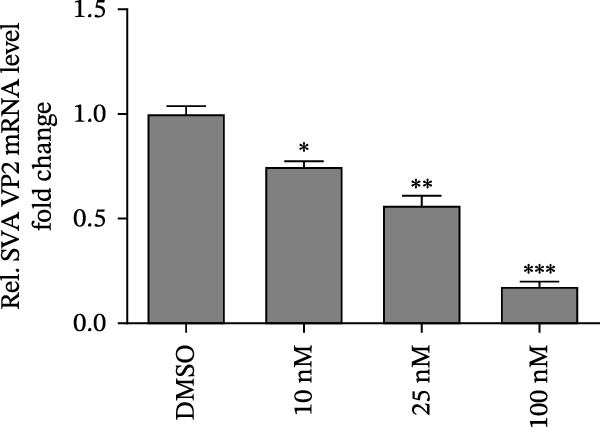
(C)

**Figure:**
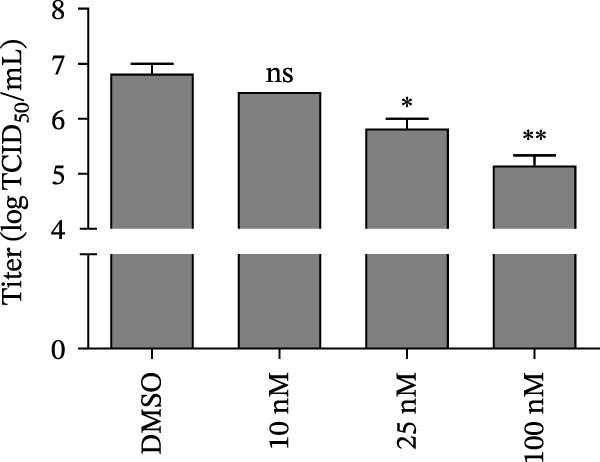
(D)

### 2.3. PMA Inhibits SVA at an Early Stage of Infection

To determine the stage at which PMA acts on SVA infection, PK‐15 cells were treated with PMA and infected by SVA follow the pattern diagram (Figure 4A). The cells were harvested for western blot, and cells were treated with PMA during infection were more resistant than those treated before, or after viral infection (Figure 4B). Visualized fluorescence was much clearer to support (Figure 4D). Meanwhile, the cells were harvested for RNA extraction and qRT‐PCR was performed for quantifying relative abundance of VP2 mRNA. Apparently, the transcription level of the virus decreased while drug treatment and viral infection simultaneously occurred (Figure 4C). We then analyzed the number of infectious viral particles by TCID_50_. PMA decreased the number of SVA particles (Figure 4E). Taken together, these data indicate that PMA limits SVA infection at the early stage.

Figure 4PMA defends against the early invasion of SVA. (A) Time‐of‐addition schematic. PK‐15 cells were infected with SVA for 1 h (0–1 h), and cells were treated with PMA (25 nM) at different hours post infection, designated pretreatment (pre), cotreatment (co), or posttreatment (post). (B) Western blot for detection of the SVA VP2 expression after pretreatment (pre), cotreatment (co), or posttreatment (post) with PMA. Similarly, changes in SVA replication were visualized with an inverted microscope. (D) Viral RNA levels were quantitated using qRT‐PCR (C), and virus yields presented as TCID_50_ per milliliter (E). The experimental data are representative of results from three independent experiments. ns, not significant;  ^∗^, *p*  < 0.05;  ^∗∗^, *p*  < 0.01.

**Figure:**
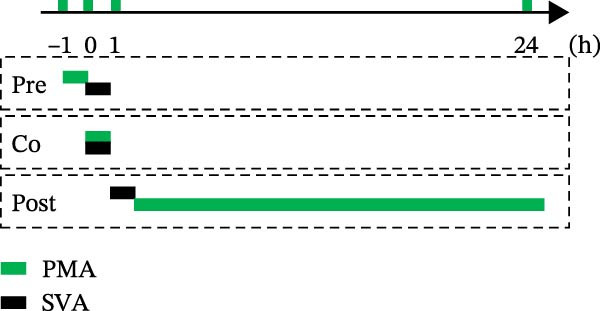
(A)

**Figure:**
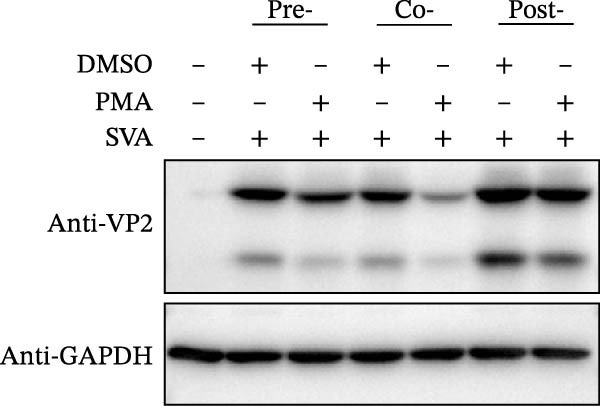
(B)

**Figure:**
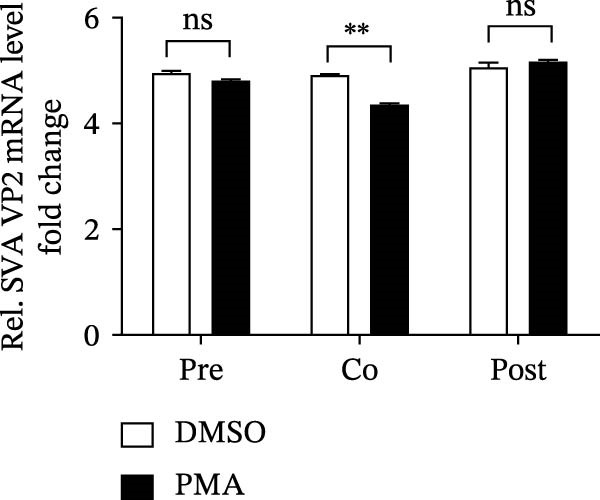
(C)

**Figure:**
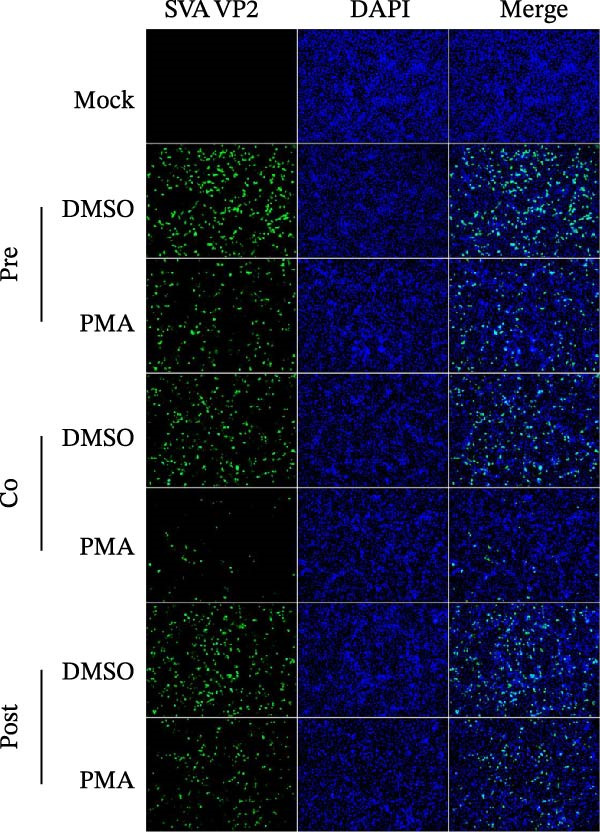
(D)

**Figure:**
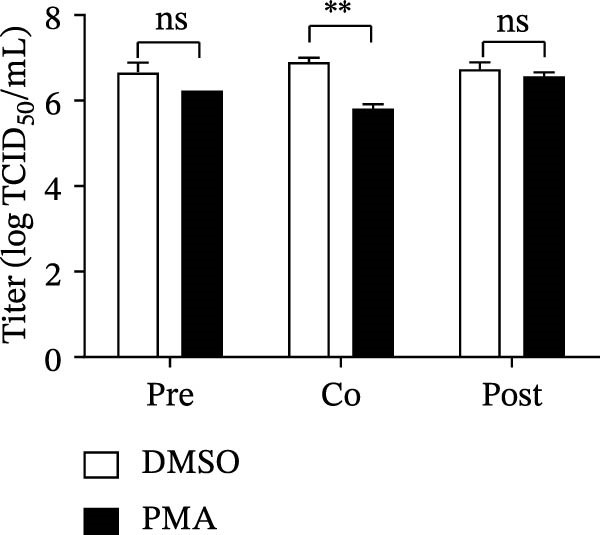
(E)

### 2.4. PMA Activates IFN Pathway and NF‐κB Signal

PMA, a mimetic of diacylglycerol (DAG), acts as an inflammatory inducer and macrophage stimulator by activating heterogeneous PKC family [23]. Moreover, PMA is a known agonist of NF‐κB pathway, and IKBKE is part of a novel PMA–inducible IκB kinase complex [24]. Therefore, we suspected that PMA restricted viral replication by activating the NF‐κB signaling cascade and IFN pathway. To check the hypothesis, cells pretreated by PMA were harvested for qRT‐PCR and western blot, respectively. IKBKE transcript abundance was upregulated by PMA, and PMA induced IKBKE protein expression (Figure 5A,B). Further experiments demonstrated that the mRNA levels of IFN‐β were upregulated when PK‐15 cells were treated with PMA (5 µM), and IFIT2, CXCL 10 and IL‐10 transcript abundance were up‐regulated when PK‐15 cells were treated with PMA (1 µM), suggesting that PMA treatment activates different immune cascades in cells (Figure 5C–F).

Figure 5PMA activates IFN pathway and NF‐κB signal. (A) IKBKE transcript abundance was upregulated by PMA. PK‐15 cells were treated with increasing doses of PMA, and then, cells were harvested for qRT‐PCR detection of IKBKE. (B) PMA induced IKBKE protein expression. PK‐15 cells were treated with different doses of PMA, and then, cells were harvested for western blot against IKBKE. ImageJ was used to quantify the level of protein, and GAPDH was used as loading control. (C–F) PMA activates IFN pathway and NF‐κB signal. PK‐15 cells were treated with increasing doses of PMA, and then, cells were harvested for qRT‐PCR detection of IFN‐β, IFIT2, CXCL10, and IL‐10. Data are expressed as the means ± SEM. ns, not significant;  ^∗∗^, *p* < 0.01,  ^∗∗∗^, *p* < 0.001.

**Figure:**
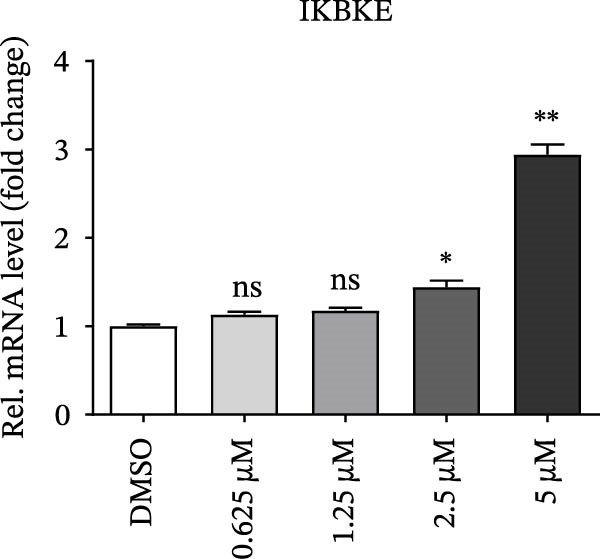
(A)

**Figure:**
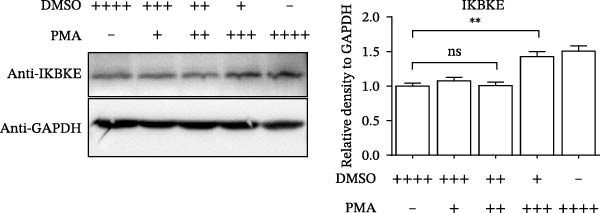
(B)

**Figure:**
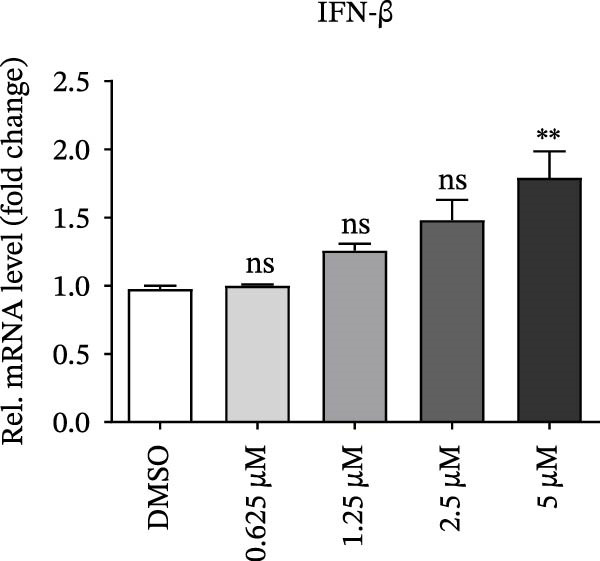
(C)

**Figure:**
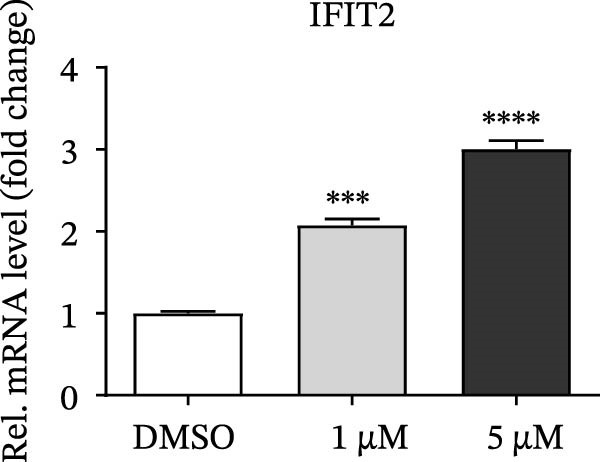
(D)

**Figure:**
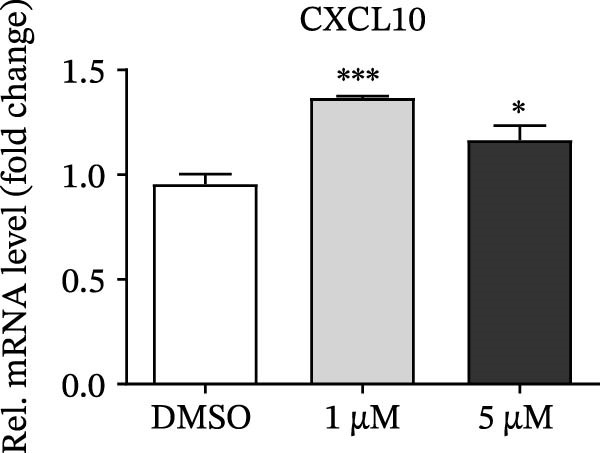
(E)

**Figure:**
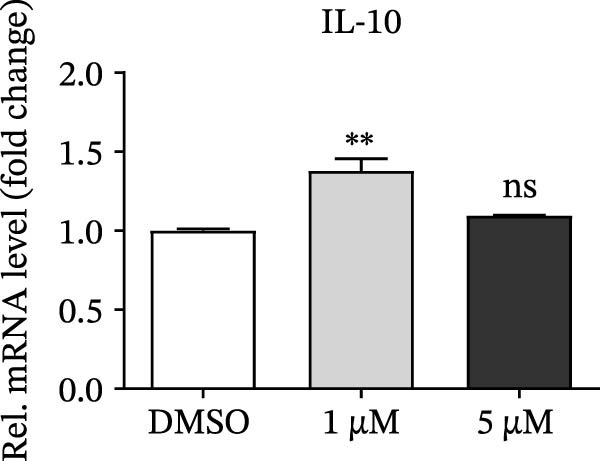
(F)

### 2.5. PMA Downregulates Viral Replication by Targeting IKBKE

As a PMA target protein, IKBKE possesses a pivotal role in coordinating the activation of IRF3 and NF‐κB in the innate immune response [25, 26]. Furthermore, overexpression of IKBKE inhibits the hepatitis C virus (HCV) replicon and partially restores IFN induction [27]. IKBKE isoform 2 (IKBKE v2) interacts with IRF7 and promotes IRF7 activation through phosphorylation and translocation of IRF7 in the presence of ubiquitin, by which the expression of IFN‐β and ISGs is elicited and viral propagation is attenuated [28]. To assess whether IKBKE similarly antagonized SVA, PK‐15 cells were transfected with IKBKE and then infected by SVA. Prospectively, IKBKE significantly diminished SVA transmission (Figure 6A). To further reinforce IKBKE as a PMA target, IKBKE‐knockout (IKBKE^−/−^) HEK‐293T cells were obtained by CRISPR/Cas9 mutagenesis (Figure 6B), and IKBKE^−/−^ HEK‐293T cells were successfully obtained (Figure 6C,D). IKBKE^+/+^ and IKBKE^−/−^ HEK‐293T cells were treated with PMA (1 µM) and infected with SVA at 0.1 MOI. At 24 hpi, the cells were harvested for western blot. PMA still had a strong antiviral effect in HEK‐293T cells, whereas the adverse effect was remedied in IKBKE^−/−^ HEK‐293T cells (Figure 6E). Similarly, cells were treated with a combination of PMA and BAY‐117082 (an inhibitor of NF‐κB), and inhibition of SVA proliferation by PMA was eliminated (Figure 6F,G). Consequently, PMA downregulates viral replication by targeting IKBKE.

Figure 6PMA downregulates viral replication by targeting IKBKE. (A) IKBKE inhibited SVA replication. pIKBKE‐flag or empty vector was transfected into PK‐15 cells. At 24 hpt, cells were infected by SVA (1 MOI) for different times. The cells were harvested for western blot. ImageJ was used to quantify the level of protein, and GAPDH was used as loading control. (B) Schematic diagram of the construction of IKBKE‐deficient HEK‐293T cells. (C) Western blot analysis of IKBKE expression in IKBKE^+/+^ and IKBKE^−/−^ cells. (D) Viability determination of IKBKE^+/+^ and IKBKE^−/−^ cells. (E) IKBKE^+/+^ and IKBKE^−/−^ HEK‐293T cells were treated with PMA (1 µM) and infected by 0.1 MOI SVA. At 24 hpi, the cells were harvested for western blot. (F) Cytotoxicity of BAY‐117082. (G) PK‐15 cells were infected by SVA and simultaneously treated with PMA (25 nM) or mixture of PMA and BAY‐117082. At 24 hpi, the cells were harvested for western blot. Experiments were performed three times. Data are expressed as the means ± SEM. ns, not significant;  ^∗∗^, *p* < 0.01;  ^∗∗∗^, *p* < 0.001.

**Figure:**
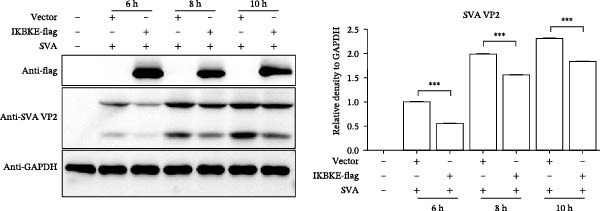
(A)

**Figure:**
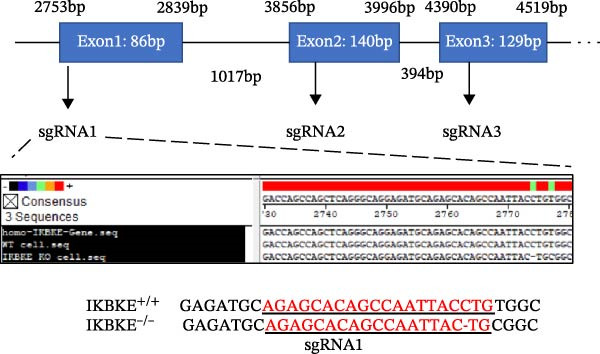
(B)

**Figure:**
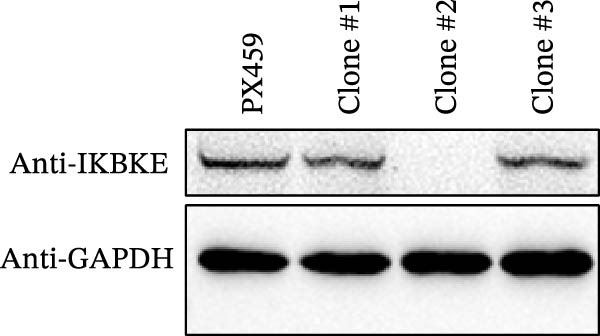
(C)

**Figure:**
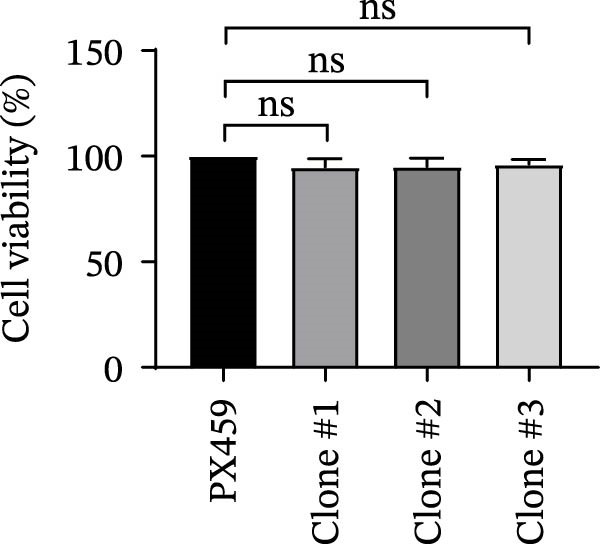
(D)

**Figure:**
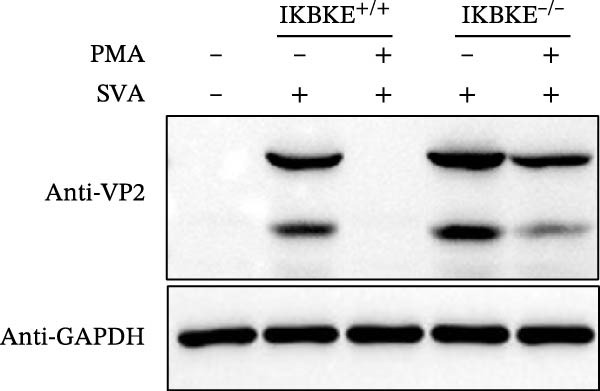
(E)

**Figure:**
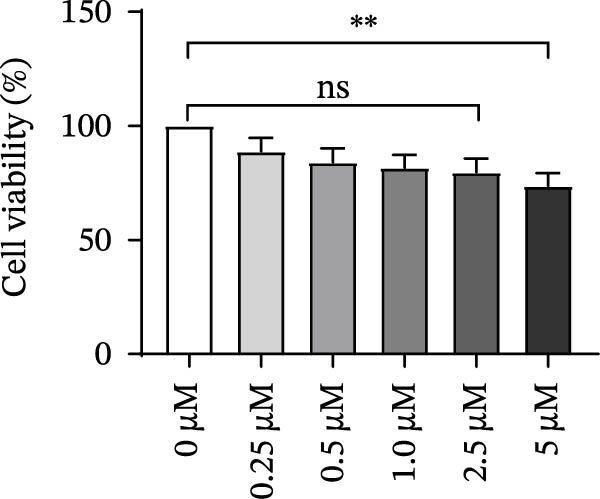
(F)

**Figure:**
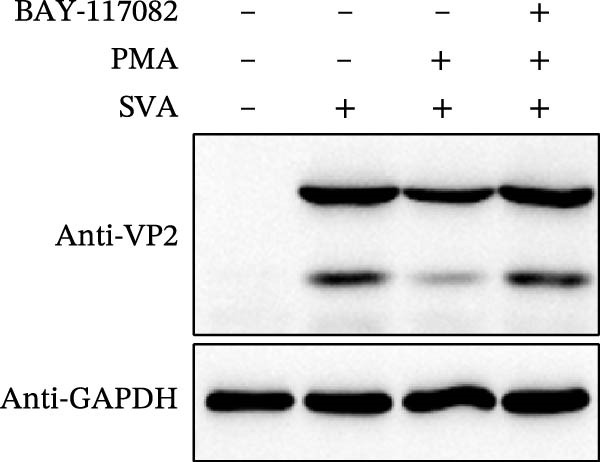
(G)

### 2.6. PMA Possesses Broad‐Spectrum Antiviral Effect on RNA Viruses

Since IKBKE antagonized multiple RNA virus infections, we speculated that the inducer of IKBKE had the semblable physiological function. To test this hypothesis, HEK‐293T cells were treated with an increasing dose of PMA and challenged by encephalomyocarditis virus (EMCV). The VP1 expression was determined via specific antibody, and rapidly downregulated in the pharmaceutical treatment group (Figure 7A). Consistent with this, the level of viral transcription and the production of viral progeny were threatened, explaining the inhibition of EMCV by PMA (Figure 7B,C). Whether PMA acted on RNA viruses other than picornavirus remained to be investigated. Next, effect of pharmaceutical treatment on porcine epidemic diarrhea virus (PEDV) replication were inspected. PEDV replication in Vero cells was gradually destroyed at both the transcription and translation levels of the virus (Figure 7D,E). Correspondingly, reduced production of virions was also observed (Figure 7F). Subsequently, we clarified that PMA impaired porcine reproductive and respiratory syndrome virus (PRRSV) replication by utilizing western blot, qRT‐PCR, and TCID_50_ (Figure 7G–I). In all, the results illuminate that PMA plays broad‐spectrum antiviral role on RNA viruses.

Figure 7PMA possesses broad‐spectrum antiviral effect on RNA viruses. (A–C) HEK‐293T cells were infected by EMCV (1 MOI) and treated with increasing concentrations of PMA (0.01, 0.1, and 1 μM). At 10 hpi, the samples were collected for Western blot (A), qRT‐PCR (B), and TCID_50_ (C). (D–F) Vero cells were infected by PEDV (0.1 MOI) and treated with increasing concentrations of PMA (0.01, 0.1, and 1 μM). At 18 hpi, the cells were collected for Western blot (D), qRT‐PCR (E), and TCID_50_ (F). (G–I) Marc‐145 cells were infected by PRRSV (0.1 MOI) and treated with increasing concentrations of compounds (0.01, 0.1, and 1 μM). At 36 hpi, the cells were harvested for Western blot (G), qRT‐PCR (H), and TCID_50_ (I). Experiments were performed three times. Data are expressed as the means ± SEM. ns, not significant;  ^∗∗^, *p* < 0.01.

**Figure:**
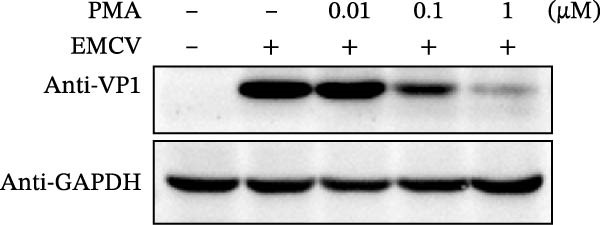
(A)

**Figure:**
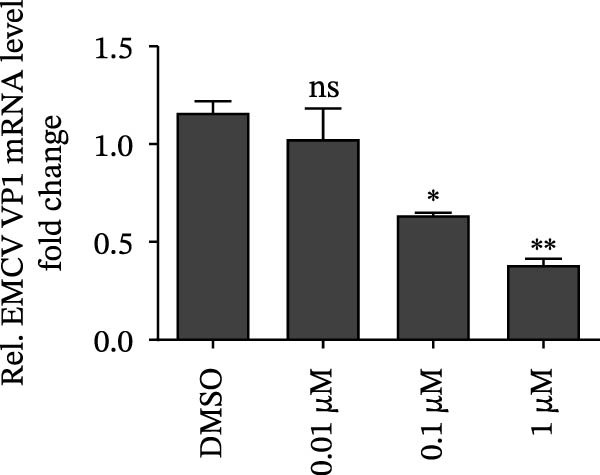
(B)

**Figure:**
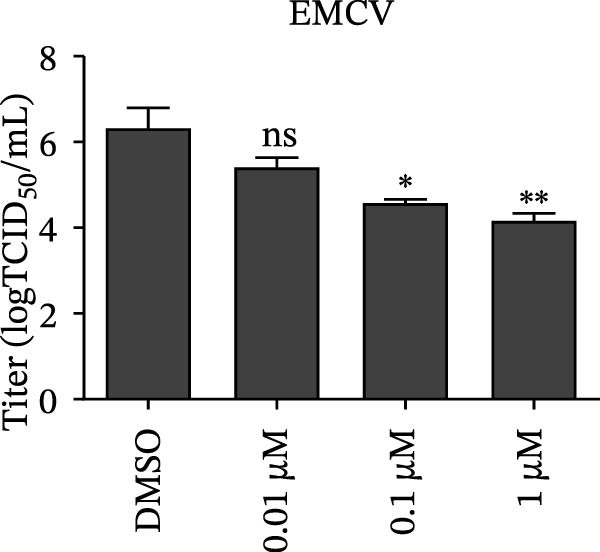
(C)

**Figure:**
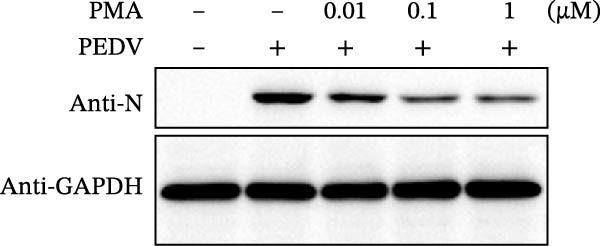
(D)

**Figure:**
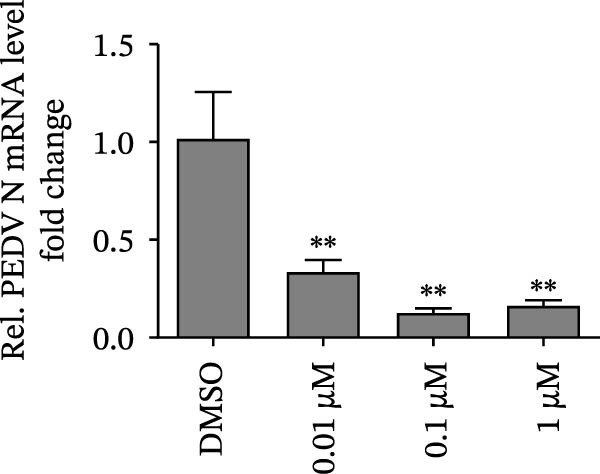
(E)

**Figure:**
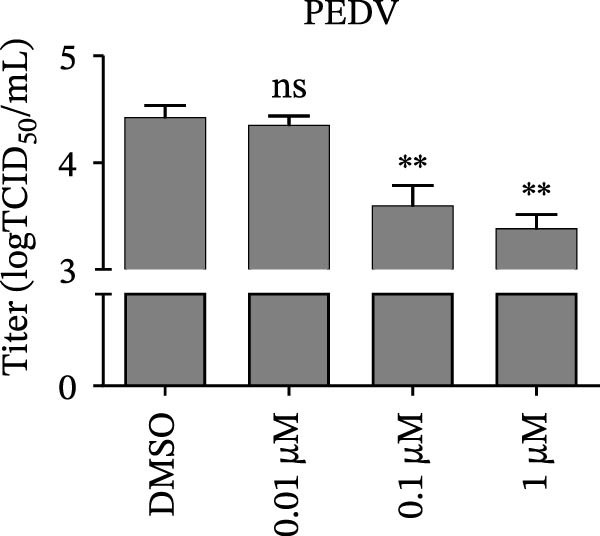
(F)

**Figure:**
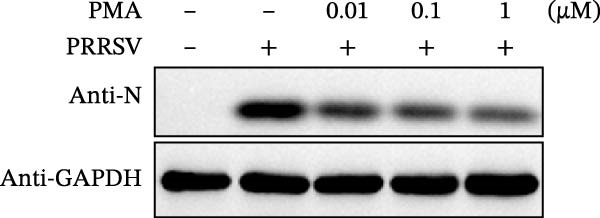
(G)

**Figure:**
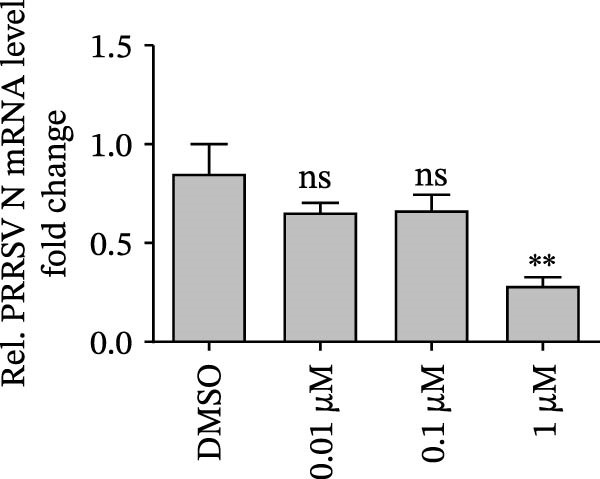
(H)

**Figure:**
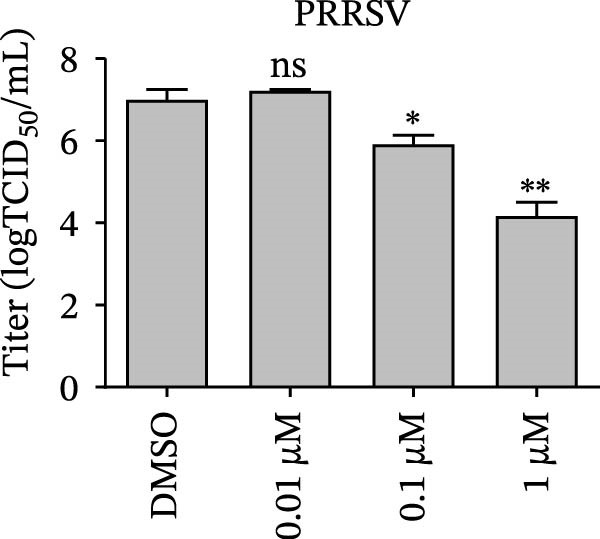
(I)

## 3. Discussion

SVA, an emerging pathogen, has caused considerable losses to the pig industry worldwide due to its similar clinical symptoms to blister diseases such as foot‐and‐mouth disease. At a critical juncture, vaccine development has encountered obstacles. To date, there is no commercially available vaccine against SVA infection. Therefore, there is an urgent need to develop alternative antiviral strategies. In study, we proposed a pharmaceutical treatment for SVA infection, and our data uncovered that PMA exhibited forceful anti‐SVA activity at an early stage in vitro. Mechanically, PMA upregulated the expression of IKBKE and induced IKBKE mediated immune responses. Remarkedly, PMA had broad‐spectrum antiviral effect on RNA viruses. It has promising therapeutic potential against SVA infection.

Phorbol myristate acetate, a mimetic of DAG, activates PKC isoforms PKC‐δ, and modulates inflammatory signaling. Our data complemented the link between PMA and viruses. The value of IC_50_ and CC_50_ of PMA showed that efficiency and security of PMA. The larger than expected SI indicates that the antiviral effect of PMA is promising to be verified in pig models. Moreover, PMA directly induces NF‐κB pathway [29]. Upon activation, the IKKβ subunit is the major responsible for IkB phosphorylation in serine residues. After phosphorylation, IkB proteins undergo ubiquitin‐dependent degradation by the proteasome, and NF‐κB is translocated to the nucleus, triggering products of pro‐inflammatory cytokines. IKBKE, the related IκB kinase homolog, also have been implicated in NF‐κB activation [26]. Meanwhile, IKBKE can be induced by PMA [24]. Herein, we hypothesized that PMA affected viral replication through an IKBKE–mediated signaling cascade. The combination of PMA and BAY‐117082 (an inhibitor of NF‐κB) eliminated the antiviral activity of PMA, which initially confirmed our hypothesis. Further study demonstrated that PMA had faint influence on SVA replication in IKBKE knockout cells.

IKBKE is involved in the regulation of a variety of viruses. Overexpression of IKBKE inhibits HCV replicon and partially restores IFN induction [27]. Likewise, IKBKE v2 attenuated EV71 propagation by IRF7 induced signals. Consistent with these reports, upregulated IKBKE expression significantly inhibited SVA replication. Based on IKBKE’s extensive inhibitory effect on viruses, our study also extends the role of PMA in replication of other viruses. PMA treatment can not only inhibit the replication of EMCV belonging to family Picornaviridae, but also inhibit the replication of PEDV and PRRSV, suggesting that PMA has a good application prospect. Correspondingly, the establishment of porcine infection model and the evaluation of the therapeutic effect of PMA in vivo need to be solved. However, even at very low concentrations, phorbol esters show toxicological effects when fed to animals. This toxicity has restricted the use of many nutritious plants and agricultural by‐products containing phorbol esters as animal feed. Therefore, researchers have extracted or inactivated phorbol esters through various chemical and physical treatments, making protein‐rich seed meal a feed resource [30]. Alternatively, by optimizing the functional groups, PMA derivatives can be developed to study their antiviral activity in vivo. To sum up, our research suggests that PMA is a promising therapeutic agent to control SVA infection in vitro.

## 4. Materials and Methods

### 4.1. Cells, Viruses, and Reagents

Porcine kidney cells, human embryonic kidney‐293T, Vero and Marc‐145 cells were cultured in Dulbecco’s modified eagle medium supplemented with 10% fetal bovine serum and 1% penicillin/streptomycin (P/S) in a humidified 5% CO_2_ incubator at 37°C. SVV‐CH‐SD (GenBank accession number MH779611), PEDV MS (GenBank accession number MT683617), the highly pathogenic PRRSV strain BB0907 (GenBank accession number HQ315835.1), and EMCV NJ08 (GenBank accession number HM641897) were preserved in our laboratory.

PMA (Phorbol 12‐myristate 13‐acetate) was purchased from Selleck Chemicals (Houston, TX, USA) and BAY 11‐7082 (HY‐13453), IκBα phosphorylation and NF‐κB inhibitor, was purchased from MedChemExpress.

DYKDDDDK‐Tag (3B9) mAb (Same as Sigma’s Anti‐FLAG M2)(M20008) was purchased from Abmart, and glyceraldehyde 3‐phosphate dehydrogenase (GAPDH) monoclonal antibody (60004–1‐Ig) was purchased from Proteintech. Goat anti Mouse IgG (H + L)(A0216) and Goat anti‐Rabbit IgG (H + L)(A0208) were purchased from Beyotime Biotechnology. SVA‐VP2, PRRSV‐N, PEDV‐N, and EMCV‐VP1 monoclonal antibodies were prepared and stored in our laboratory.

### 4.2. Screening of a Natural Product Library

A library of 112 small molecule (FDA‐approved) was purchased from Selleck Chemicals (Houston, TX, USA). The small molecule compounds were stored as 10 mM stock solutions in DMSO at −80°C. The high‐throughput screening (HTS) process for the libraries is shown in Figure 1A,B. PK‐15 cells were pretreated with 10 µM compound or DMSO (1 µL) for 1 h and then infected with SVA (1 MOI) or mock infected for 1 h. Cells were then washed with PBS, and then, incubated for another 24 h in medium containing 10 µM compounds, the percentage of inhibition was calculated by IFA and western blot assay.

### 4.3. Cell Viability Assay

PK‐15 cells were seeded in 96‐well plates until the cell density was 100%, and then, PMA with final concentrations of 1–250 μM were respectively added to columns A1 to A11 in eight replicates. DMSO was used as solvent control. The treated cell culture plates were incubated at 37°C for 48 h. According to the instructions, the liquid in the wells was removed, and 100 μL CCK8 working solution was added to each well, and incubated at 37°C in the dark for another 2 h. Cell viability (%) was calculated by measuring OD_450_ value. The CC_50_ of the other three compounds (raddeanin A, mubritinib, and polyphyllin II) was determined as above.

### 4.4. SVA Infectivity Inhibition Assay

PK‐15 cells were seeded in 96‐well plates until the cell density was 100%, and then PK‐15 cells were pretreated with 10 µM compound or DMSO (1 µL) for 1 h and then infected with SVA (1 MOI) or mock infected for 1 h. Cells were then washed with PBS, and then incubated for another 24 h in medium containing 10 µM compounds. The percentage of inhibition was calculated by IFA. Fluorescence intensity was quantified by Image J software. The 50% inhibitory concentration (IC_50_) curves of the four compounds against SVA were plotted by GraphPad Prism 8.0 software.

### 4.5. Indirect Immunofluorescence Assay

The collected cells were fixed with 4% paraformaldehyde, incubated at room temperature for 15 min, and then, washed three times with PBS. The membranes were then permeabilized with 0.1% TritonX‐100 for 20 min at room temperature. After washing, monoclonal antibody against SVA VP2 protein (1 : 1000) were added for 1 h at 37°C. After another washing, 100 μL FlexAble CoraLite488 Antibody Labeling Kit for Mouse IgG1 was added to each well and incubated at 37°C for 45 min. The cells were washed with PBS for three times, and then, were stained with DAPI at room temperature for 3 min. The fluorescence intensity was observed under inverted fluorescence microscope.

### 4.6. Western Blot Assay

The cells were collected and lysed with RIPA lysis buffer (Beyotime, China) on ice for 15 min. Protein samples containing equal amounts were separated by 10% SDS‐PAGE and transferred to a nitrocellulose membrane. The transferred nitrocellulose membrane was incubated in PBST buffer containing 5% skim milk at room temperature for 2 h. After washing, the nitrocellulose membrane was successively bound to the specific primary antibody and the corresponding secondary antibody. Bound proteins were visualized with a Tanon 5200 chemiluminescence imaging system (Tanon, China).

### 4.7. RNA Extraction and Real‐Time Fluorescence Quantitative PCR

According to the instructions, total intracellular RNA was extracted using E.Z.N.A. Total RNA Kit, and then, PCR system was prepared and programmed to detect gene expression by using reverse transcriptase HiScript Q RT SuperMix for qPCR. The relative mRNA expression levels were statistically analyzed by 2^−*ΔΔ*Ct^.

### 4.8. Viral Titration

The harvested cell suspension was diluted at a tenfold ratio. There were eight repeat holes for each dilution and a negative control group. After incubation at 37°C for 1 h, the medium was replaced with 2% DMEM. Viral titers were determined using endpoint dilution analysis at 5 days postinoculation (dpi). Viral titers are expressed as TCID_50_, calculated using the Karber method.

### 4.9. Generation of IKBKE^−/−^ Cells

CRISPR/Cas9 genomic editing for gene deletion was performed as previously described [31]. Three sgRNA sequences were designed using an online CRISPR design tool (Benchling, https://www.benchling.com/crispr). HEK‐293T cells were constructed to stably express Cas9 and sgRNAs from a PX459 vector by selection with puromycin (10 µgmL^−1^). Single clones were obtained by serial dilution.

### 4.10. Statistical Analysis

All data were analyzed with GraphPad Prism 8.0 using one‐way analysis of variance (ANOVA), two‐way ANOVA, or Student’s *t*‐test. Results are presented as mean ± s.e.m. *p*‐Values are indicated using asterisks:  ^∗^ *p*  < 0.05,  ^∗∗^ *p*  < 0.01, and  ^∗∗∗^ *p*  < 0.001.

## Author Contributions

**Junfang Yan**: conceptualization, data curation, formal analysis, methodology, software, writing – original draft, writing – review and editing. **Yanni Gao**: conceptualization, methodology, writing – original draft, writing – review and editing. **Chengyi Guo and Yubei Dong**: methodology, software. **Ping Jiang**: conceptualization, funding acquisition, methodology, project administration, resources. **Juan Bai**: conceptualization, formal analysis, funding acquisition, resources, writing – review and editing.

## Funding

This work was supported by the National Key Program of Research and Development of China (Grant 2022YFD1800802), the China Agriculture Research System of MOF and MARA (Grant CARS‐35), the Jiangsu Independent Innovation Fund Project (Grant CX (22)1011), and a grant from Jiangsu Province (Grant PAPD).

## Conflicts of Interest

The authors declare no conflicts of interest.

## Data Availability

The data that support the findings of this study are available upon request from the corresponding author. The data are not publicly available due to privacy or ethical restrictions.
